# “No Regrets” Purchasing in a pandemic: making the most of advance purchase agreements

**DOI:** 10.1186/s12992-022-00851-3

**Published:** 2022-06-17

**Authors:** Ian Thornton, Paul Wilson, Gian Gandhi

**Affiliations:** 1 UNICEF Supply Division, New York City, NY USA; 2 Mailman School of Public Health, Columbia University, New York City, NY USA

**Keywords:** Pandemic preparedness and response, Public health emergency response, Health product purchasing, Vaccine equity, R&D incentives, COVID-19, Vaccine, Therapeutics, Diagnostics, PPE, Medical countermeasures

## Abstract

“No regrets” buying – using Advance Purchase Agreements (APAs) – has characterized the response to recent pandemics such as Avian flu, Zika Virus, and now COVID-19. APAs are used to reduce demand uncertainty for product developers and manufacturers; to hedge against R&D and manufacturing risks; and to secure availability of products in the face of spiking demand. Evidence on the use of APAs to buy vaccines, medicines, diagnostics, and personal protective equipment during recent pandemics illustrates how these contracts can achieve their intended objectives for buyers. But, transferring risk from suppliers to buyers - as APAs do - can have consequences, including overbuying and overpaying. Furthermore, the widespread use of APAs by high-income countries has contributed to the striking inequities that have characterized the Swine flu and COVID-19 responses, delaying access to vaccines and other supplies for low- and middle-income countries (L&MICs).

We identify seven ways to address some of the risks and disadvantages of APAs, including adoption of a global framework governing how countries enter into APAs and share any resulting supplies; voluntary pooling through joint or coordinated APAs; a concessional-capital-backed facility to allow international buyers and L&MICs to place options on products as an alternative to full purchase commitments; greater collection and sharing of market information to help buyers place smarter APAs; support for a resale market; building in mechanisms for donation from the outset; and transitioning away from APAs as markets mature. While a binding global framework could in theory prevent the competitive buying and hoarding that have characterized country/state responses to pandemics, it will be very challenging to put in place. The other solutions, while less sweeping, can nonetheless mitigate both the inequities associated with the current uncoordinated use of APAs and also some of the risks to individual buyers.

Analysis of recent experiences can provide useful lessons on APAs for the next pandemic. It will be important to keep in mind, however, that these contractual instruments work by transferring risk to the buyer, and that buyers must therefore accept the consequences. In the spirit of “no regrets” purchasing, having bought what hindsight suggests was too much is generally preferable to having bought not enough.

## Background

In emergency management, there is a principle known as “no regrets”: the idea that in an unpredictable crisis, we should overprepare rather than ‘wait and see’ [1]. No regrets buying has characterized the current pandemic response the world over, whether it be for Personal Protective Equipment (PPE) or for COVID-19 vaccines. No regrets purchases often took the form of Advance Purchase Agreements (APAs): deals in which a purchaser commits in advance to buying goods, even if they may no longer be needed when they become available. Sometimes the goods are actually paid for in advance, and sometimes the purchaser is not even sure they will get the goods at all.

It is two years after the first APAs were signed for COVID-19 vaccines. As the world looks back, and as market actors grapple with the consequences of unprecedented deal making in the biopharmaceutical sector, we examine the use of APAs in recent pandemic response efforts: what they are; why they are used; what their consequences have been; and what we can learn from this and other pandemics to make better use of them.

## What are APAs?

APAs are also described as Advance Purchase Commitments (APCs) or Advance Price or Purchase Commitments (APPCs) [2] and can take a variety of forms. In essence, they are binding commitments to individual suppliers to purchase not-yet-available products, if certain conditions are met, whether or not expected demand for the products materializes and whether or not the products are still needed. In both popular and academic discourse, they are sometimes conflated with Advance Market Commitments (AMC) [3], which are commitments to potential suppliers as a group rather than to individual firms (Table 1).

**Table 1:** APAs and AMCs

	**Advance Purchase Agreements**	**Advance Market Commitments**
Description	Buyer agrees to purchase a fixed volume of a specific product from a specific seller at an agreed price, often in advance of firm demand materializing and sometimes before the product has come to market or manufacturing capacity has been established	Buyer commits in advance to purchase a volume of qualifying products at an agreed price or to subsidize purchases of these products by eligible countries
Examples	Buyer A commits to purchasing 10 million units of Product X from firm M at $10/unit, if the product is approved by specified regulatory authorities	a) Buyer B commits to purchase 10 million units of products of type Y at $10 from any firm with a qualifying product, OR b) Buyer C [in this case either a Proxy buyer (e.g., international organization buying on behalf of multiple countries) or donor] commits to “top up” to $10 all purchases of qualifying products of type Z by eligible countries paying a lower price, up to 10 million units
Uses	Incentivize R&D or supply by reducing demand risk to suppliers, notably in disease outbreaks. Reduce supply risk to buyers by tying up supply	a) Incentivize R&D or supply by reducing *aggregate* demand risk, while retaining a competitive dynamic, OR b) Incentivize R&D or supply by creating a subsidized market without insulating firms from demand uncertainty

APAs reduce demand risk to suppliers by (i) guaranteeing the price to be paid, (ii) guaranteeing the volume to be purchased, or (iii) a combination of the two – amounting to a guarantee of revenue. The ‘upfront’ nature of an APA is usually provided via a legally binding commitment [4, 5], a full or partial payment upfront, or both [6]. When the products in question are still being developed, APAs typically include some form of conditionality, such as achievement of licensure by a stringent regulatory authority, reaching some other development milestone, or meeting specific technical criteria. In some cases, the contract may also be triggered by a pre-defined event, such as declaration of a Public Health Emergency of International Concern (PHEIC) by WHO [7].

In contrast, AMCs are commitments to suppliers as a group rather than bilateral contracts with individual firms. In one form, they aim to incentivize suppliers by promising to buy an agreed quantity of qualifying products at a fixed price without committing specific volumes to particular suppliers. This kind of AMC, like APAs, would in theory protect suppliers from the aggregate demand risk that is such an important feature of pandemics. Some have argued that AMCs of this type would be more appropriate than APAs for pandemic vaccines, either from the beginning or for second- and third-generation vaccines, but these proposals have not been taken up so far. [8, 9] (See Sect. 8 below.) A second type of AMC, exemplified by the Pneumococcal Conjugate Vaccine (PCV) AMC, creates a subsidized market for, but does not insulate suppliers entirely from, demand uncertainty, and is less relevant to pandemic markets. It is important to note in the context of COVAX [10] that the “COVAX AMC” is not technically an AMC, but a group of APAs. It was called an AMC in part because key donors were familiar the term from their investment in the PCV AMC and had an existing budget line for “an AMC”, facilitating their investment in COVAX.

## Why use APAs?

APAs are used sparingly in the normal course of global health procurement, as product developers and suppliers accept some degree of demand uncertainty as an established business risk. But disease outbreaks, especially large-scale events like the COVID-19 pandemic, introduce uncertainties of a different order, for both suppliers and buyers. At the start of an outbreak, it is very difficult to predict how severe the outbreak will be, which countries it will affect, how long it will last, and what kinds of products will be needed. As a result, suppliers considering developing new products—or deciding how much to scale up manufacturing of existing products—face a very real risk that the outbreak will be over be before their products can come to market or that hoped-for demand will not materialize. Given this epidemiology-derived uncertainty, many firms may decide not to invest in product development or expanded capacity unless this demand risk is mitigated, at least in part, including by some form of purchase commitment. The most fundamental value of APAs, then, is to address three related market objectives:

- 1. Reduce demand uncertainty/risk for product developers or manufacturers and thus encourage product development and supply [11].
  For buyers, APAs are also a way to.
- 2. Secure availability of a desired product in the face of high demand [12].
  Since there is no guarantee that a particular product will come to market, buyers also use multiple APAs for a portfolio of products to.
- 3. Hedge against R&D and manufacturing risk [13].

## What’s the utility of APAs?

### APAs are a useful, and even necessary, tool for buying in epidemics and pandemics

Below are a series of examples of the use of APAs, grouped by the three objectives described above:

### Reducing demand uncertainty and accelerating product development and manufacturing

- In the early 2000’s, the threat posed by H5N1—‘Avian flu’[14] catalyzed a focus on pandemic preparedness, and APAs became widely used to secure vaccines for the subsequent pandemic influenza threat, H1N1—‘Swine flu’. Within the European Union, 16 countries had an APA in place [15]. This meant manufacturing capacity was set aside, and the first manufacturers were able to supply H1N1 vaccines around three months after the declaration of a PHEIC. However, while these APAs worked well for their (mostly High-Income Country (HIC)) buyers, they left insufficient manufacturing capacity for WHO and non-APA holders, at least during the first year of the pandemic [16].
- In 2015–16, the future course of the Zika Virus (ZIKV) epidemic was uncertain, making it difficult to project need and demand for ZIKV Point-of-Care (POC) Rapid Diagnostic Tests (RDTs) [17]. Epidemiological risk (impacting demand risk), along with lack of clarity on desired product characteristics, created a risky environment for potential RDT developers. With $10 million in funding from The United States Agency for International Development (USAID), UNICEF created a market-wide guarantee and awarded APAs to a range of POC RDT developers with promising prototypes. These were conditional on the products meeting pre-defined standards of sensitivity and specificity, as measured by third-party laboratories. Three tests from two manufacturers passed the validation step and UNICEF committed to buying ~ 1.2 million tests over a 3-year period [18]. Participating developers strongly suggested that the initiative influenced their decisions on R&D investment and product profiles [19]. However, this example illustrates the buyer-side risks as well as the benefits of APAs, as there has not yet been a major new ZIKV outbreak to drive ongoing demand for these tests.
- The first and largest APAs signed with COVID-19 vaccine manufacturers incentivized them to scale up production even before trial results were available. Johnson and Johnson/Janssen, Moderna, and Pfizer-BioNTech received APAs amounting to $11.92 billion to produce 700 million doses for the US by February 2021 [20]. By way of comparison, the total global influenza vaccine market (across all countries and firms) pre-COVID-19 was estimated to be worth $5–6 billion [21]. While the development of COVID-19 vaccines has been the fastest in history, it is difficult to know to what extent the APAs accelerated progress by reducing demand uncertainty, as large-scale push funding (to subsidize R&D costs), overlapping clinical development processes (e.g., overlapping preclinical and phase II/III trials rather than running these sequentially) [22, 23] and compressed regulatory pathways also contributed [24]. But it seems reasonable to assume that the prospect of very large and guaranteed markets influenced the scale of firms’ commitments.
- With COVID-19 vaccines came the need for vastly increased syringe production, and a new size of syringe. APAs were necessary in the case of the new 0.3 mL Auto Disable syringes: L&MIC demand was almost exclusively dependent on US Government donations of the Pfizer-BioNTech doses, and these syringes had no other obvious purpose – they could not be stored and used in routine immunization. While the Pfizer-BioNTech vaccine has now seen high uptake, this was not a given at the time: the US Government volumes were pledged in increments, and country-level demand was very uncertain given the complex ultra-cold-chain requirements for this vaccine.

### Securing supply

- At the start of the COVID-19 pandemic, buyers of all types scrambled to secure supplies of PPE – at the time the only available medical countermeasure and first line of defense. Demand quickly outstripped supply and prices skyrocketed. To exacerbate the situation, most of the world’s PPE production was located in China and the raw materials were heavily concentrated in China and India. These countries were in lockdown or were imposing export restrictions, reducing supply and increasing lead-times for the rest of the world. UNICEF, like other buyers, deployed APAs [25] to reduce risk for manufacturers who wanted to invest in expanding production capacity; to reserve their place in the production queue; and secure access to supply. Supply did materialize – in fact, the initial frenzy and high prices encouraged so many new firms to enter this market that supply soon outstripped demand and prices fell to pre-pandemic levels [26]. Some buyers who signed APAs at the top of the market ended up paying more than they had to, in exchange for limited acceleration in access.
- In June 2020, a trial showed that Dexamethasone (Dexa) could reduce COVID-19 mortality for some patients [27]. Experience with PPE a few months earlier suggested that there might be a rush on Dexa supply. UNICEF, in partnership with UNITAID and the Access to COVID-19 Tools (ACT) Therapeutics Accelerator, deployed an APA to secure some of the available Dexa supply, even before potential demand from L&MICs was well understood [28]. Supply was secured at an affordable price and UNICEF was able to ship Dexa to 37 countries between August 2020 and November 2021 [29]. Similarly, over 2021, UNICEF deployed seven APAs for 26,000 oxygen concentrators, 250 ventilators and 90 Pressure Swing Adsorption plants to generate oxygen [30]. At the time, UNICEF faced fierce buyer competition and manufacturers had limited production – the combination of which created long lead-times for access. UNICEF was able to reduce lead-times by around 50% for most of the oxygen products. Given demand outstripped supply but and the range of alternative uses of these products outside of COVID-19 (e.g., in treatment of pneumonia), the risk of overbuying oxygen products was low. UNICEF expects to have deployed all stocks purchased under APA by the end of 2022.

### Hedging against R&D risks

- Most buyers of COVID-19 vaccines put down APAs for multiple products, based on different technology platforms, sourced from different manufacturers – and in some instances from multiple manufacturing sites—in order to increase the chance that they would have access to at least one vaccine if others failed to come to market. The extraordinary R&D success rate with COVID-19 vaccines, however, means that the global markets for COVID-19 vaccines is now characterized an unprecedented variety of products, but also significant oversupply [31–34].

## APA risks for buyers

### As they work by transferring demand risk, APAs come with some unavoidable risk of overbuying or overpaying. This intrinsic risk should be understood and accepted

Fundamentally, APAs work by transferring risk from supplier to buyer. They are most useful when very high demand uncertainty may deter suppliers from developing or preparing to manufacture a needed product. But the downside of assuming this risk is that the buyers may end up purchasing more product than they need or paying more than necessary for the needed product when demand does not materialize. Similarly, when buyers enter into multiple APAs to hedge against R&D failure, they must accept the possibility that if more products than expected come to market they will end up buying more than they need, including specific products that are less desirable than others. These risks can be mitigated but cannot be avoided altogether.

The Ebola and ZIKV outbreaks provide good examples of the first risk, that purchasers, working with information available at the time, can over-estimate demand and/or its longevity, and be left buying supplies that, in hindsight, were not needed. Both outbreaks waned after APAs were put in place and demand plummeted [35, 36], highlighting the centrality of epidemiological risk in demand risk and therefore APA use. The case of COVID-19 vaccines is more nuanced in this regard. Overall, it can hardly be said that buyers overestimated the severity of the outbreak or the need for a vaccine early on, when the first large APAs were signed. But as we enter the third year of the pandemic, the picture is less clear. The pandemic is not over and Omicron variants are still infecting and killing people. However, many populations may have acquired substantial immunity through infection as well as vaccination [37]. This, combined with pandemic fatigue and evidence that current vaccines only partly prevent transmission of the virus, means that purchasers are seeing demand ‘soften’ for COVID-19 vaccines at the very time that supply to L&MICs is increasing fast. Moreover, demand is higher for some COVID-19 vaccines than others. This illustrates that overbuying can happen when there is *timing* mismatch between demand and secured supply.

Overbuying can also materialize if an international proxy buyer such as UNICEF or COVAX, buying on behalf of multiple L&MICs, cannot fully ascertain purchasing patterns via other channels of access. During the COVID-19 pandemic, most L&MICs have tried meet their vaccine needs via multiple channels, including COVAX, the African Union’s African Vaccine Acquisition Trust (AVAT), bilateral deals, or third-party donations. Given limited communication between buyers and uncertainties as to when doses from each channel would materialize, the risk of oversupply to particular countries is multiplied. In this instance, as countries are likely to privilege doses obtained through bilateral deals or donation, it is the international proxy buyers that may be left with unwanted doses and the accompanying financial exposure.

Similarly, when purchasing Dexa, UNICEF and UNITAID were aware that the product was already used widely for other applications but had limited information on available stocks in L&MICs. What is more, these countries already had their own channels for accessing this drug from local and international pharmaceutical companies. In part because of the paucity of COVID-19 testing, the surge in demand and market shortages that were predicted for Dexa did not materialize. As a result, UNICEF has so far been able to use some but not all of the stocks that it procured.

Lastly, overbuying can occur if international proxy buyers fail to anticipate evolution in product preferences or under-estimate blockages elsewhere in the system. COVID-19 vaccines illustrate well how product preferences can shift with changing evidence/perceptions on safety profiles (Astra Zeneca, Johnson & Johnson/Janssen vaccines), efficacy (Sinovac Biotech, Sinopharm vaccines), protection against new variants (all COVID-19 vaccines products). In addition, an early mover advantage can solidify preferences — low demand for protein subunit COVID-19 vaccines is probably explained in considerable part by reticence to shift away from products already in use. Furthermore, an APA holder is always just one part of a broader system: regulatory approval in recipient countries, normative guidance on appropriate use of products, delivery support and many other elements are needed to create viable demand and a functional supply chain for COVID-19 vaccines to materialize, adding to the risk assumed by the buyer.

A related risk to buyers is that they pay too high a price. This can occur because APAs are usually signed when supply is constrained (or not yet available at all), a buyer’s willingness to pay is high, and the buyer has underestimated the elasticity of supply. If supply increases more rapidly than anticipated, either because existing suppliers scale up or new suppliers enter the market, buyers may end up paying more for products than they would have paid if they had waited for the market to evolve. Much of this risk stems from ‘information asymmetry’: sellers (product developers and manufacturers) are in general better informed about their supply capacities, timelines, and costs than buyers. As with the risk of overbuying, some risk of paying more than ultimately proves necessary can be seen as the price of supply security.

**Fig. 1:**
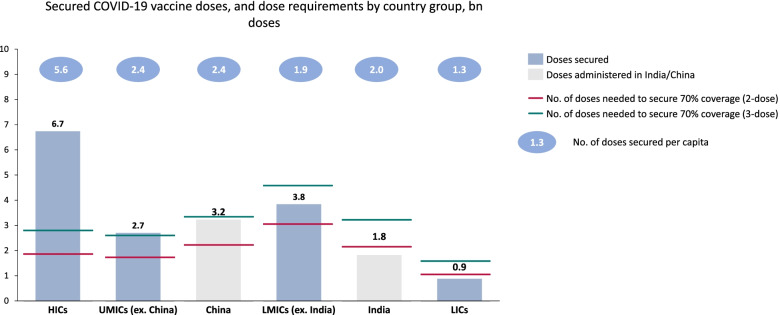
Secured COVID-19 vaccine doses, and dose requirements by country group. The data on secured doses are from the IMF-WHO COVID-19 Vaccine Tracker [39]. For China and India, the authors chose to use administered doses from Our World In Data [40] rather than secured doses, because the underlying source for the IMF dashboard (UNICEF data) is less comprehensive in tracking Indian and Chinese government deals with their domestic manufacturers. The dose requirements reflect the WHO strategy of 70% coverage with two doses, or, if including boosters, three doses. Each calculation includes 10% wastage

The case of COVID-19 vaccines illustrates well the risks of buying too much as a result of placing multiple APAs. All buyers, and particularly those in HICs, over-estimated the R&D risk for COVID-19 vaccines (or, equivalently, under-estimated the chances that particular products would come to market), and so overbought. For example, *by August 2020*, before any vaccines had been licensed, some HICs had already signed APAs amounting to about five doses per person, enough to fully vaccinate everyone in the country more than twice over [38]. Figure 1, below, shows the purchasing of COVID-19 vaccines by country groups, relative to need and per population.

## APA risks for suppliers

Although this is not the focus of this article, it should be noted that APAs present some risks for suppliers. The most important risk is that they may end up being obligated to sell their product at too low a price. This can happen if they underestimate their costs, underestimate demand, or overestimate supply from other sources. Indeed, if only one or two COVID-19 vaccines had come to market, desperate buyers would likely have been willing to pay considerably more than the prices agreed in APAs. This scenario illustrates the importance of being able to enforce APAs and prevent sellers from diverting supply to new buyers offering higher prices.

## Equity Implications of APAs

### APAs can contribute to inequitable access by allowing HICs to tie up supply in advance

The use of APAs is not inherently inequitable. However, HICs tend to have greater buying power than L&MICs or international buyers like UNICEF, and ability to move quicker too. As a result, HICs can tie up supply and secure their places at the front of the line to access limited supply. This appears to be happening again in the response to the current monkeypox outbreak. As long as states act primarily in their own interests, concern over scarcity will prompt efforts to secure supply, and each such initiative will push others to follow suit for fear of being left out. Those L&MICs with least ability to pay miss out, or come last, or are reliant on donations from HICs, even if their need might be considered greatest.

Ultimately, the equity challenge can be resolved definitively only by an enforceable framework for global collective action. In the absence of such a framework some of the other measures described in the next section can partially—but only partially—mitigate the equity consequences of APAs as deployed by states acting in their own interests.

Despite these concerns, it is worth emphasizing that the development and production of vaccines and other products needed in disease outbreaks are global public goods, and APAs, by reducing demand uncertainty for product developers and manufacturers, can help ensure that these products come to market. It can be argued that without APAs some of these products would not exist *for anyone,* either the deep-pocketed buyers with the financial resources to deploy APAs early, or others who are eventually able to obtain access to these products. This highlights an important, though not-straightforward, time element to equity too. Does equity mean truly simultaneous access by both those who agreed and/or paid in advance and those who did not? How many ‘places in the queue’ is it acceptable to take? Or how much tying up of supply is acceptable before opening up supply to those who placed later deals later, or none at all? These are questions that must be grappled with in any future Pandemic Preparedness & Response (PPR) architecture (within which future APAs will be deployed).

## Mitigating the disadvantages associated with APAs

### Several possible measures could help to mitigate *both* the inequities stemming from the use of APAs and the risk of overbuying to individual buyers

Here we present seven ideas for how APAs could be designed, used, or governed to better mitigate the two linked risks we have outlined – inequities and overbuying.

### Treaty/convention

A global framework governing pandemic response, perhaps embodied in a treaty or convention, could guide how countries enter into APAs and share the resulting supplies. This is part of a proposal put forward by the European Union and agreed by the World Health Assembly in December 2021 [41, 42] although there are also arguments against such a pandemic treaty [43, 44]. The analogous WHO Pandemic Influenza Preparedness (PIP) Framework created the basis for sharing of influenza virus samples and access to vaccines and could serve as a template to address equity of access to medical countermeasures for other pandemics. However, despite years of negotiation, the PIP Framework remains a narrow and non-binding instrument [45]. Whatever the prospects for a pandemic treaty, it could in theory bring about more equitable access to pandemic vaccines and other medical countermeasures by requiring buyers to act in concert, including by pooling their resources; by limiting the extent to which those with the greatest financial capacity could tie up supply through APAs; or by requiring them more rapidly or more fully share what they had purchased. A treaty might also reduce the risk of overbuying, with buyers of all types knowing they can depend on supply from others, and because they might be compelled to share their supply with others.

### Voluntary pooling

Short of a binding treaty, greater use of voluntary pooling could mitigate inequity and/or overbuying. Buyers could work together to place multiple APAs (to hedge R&D or manufacturing risks) and agree to share the products obtained through these agreements. This cooperation could take different forms, including joint deals, in which multiple buyers pool their resources and sign joint contracts with suppliers; and aligned deals, in which different buyers make deals with different manufacturers but agree to share products. One could even envisage a two-tier pooling system, where UNICEF, for example, aggregated demand and reduced transaction costs for a group of L&MICs (as it already does for many other products) and then was itself one buyer in a bigger pool.

Pooling is not perfect: those outside the pool are (by definition) excluded from access, so pooling could actually increase inequity in some situations. Furthermore, legal agreements to share doses are only so effective in the face of sovereign government-imposed export bans, for example. Pooling was the idea behind COVAX, although it was somewhat overtaken by the vaccine-nationalist responses that characterized the start of the pandemic [46]. The European Commission, however, successfully used pooling. They placed APAs with eight manufacturers on behalf of participating member states [47], and whilst only five products have come to market, they had enough supply for 70% of their population by mid July 2021.

Similarly, pooling should reduce overbuying, though this is not guaranteed. As we have seen with COVID-19, striking R&D pipeline success can lead to oversupply. Or, the uncertain duration of protection might lead a pool to double down and buy booster doses that are then not needed. Disentangling strategically sensible and morally justifiable buying versus (unnecessary/unwarranted) overbuying can be very difficult, both in the moment and in hindsight.

### Options fund/facility

An international fund or ‘facility’ might allow international buyers and L&MICs to place ‘call options’ for products rather than entering into their own APAs with suppliers. In other markets, a ‘call option’ gives a buyer a right to buy a pre-specified product at a later date at a pre-specified price. For this flexibility (the right but not the obligation to buy) they pay a ‘premium’ [48]. It might be possible to establish a fund that invests in epidemic-relevant products and then sells the rights to buy these if needed to L&MICs or international organizations. If the epidemic faded out quickly (as ZIKV did), the option buyers would lose their premiums and the facility its investment (perhaps an APA, perhaps some other kind of deal). If the epidemic lasted, the buyers would call their options and receive their products. In parallel, a donor could subsidize premiums, product purchase, or both, for countries that could not otherwise afford them.

Many questions remain about such a scheme. Given the great uncertainty associated with epidemics, would the premium have to be so high as to limit the attractiveness of options? What kinds of deals would the facility itself place with suppliers and how would they coexist with deals placed by countries outside the facility? Who would capitalize the facility and how would it relate to COVAX or a successor initiative? Nonetheless, a mechanism of this kind could lower upfront costs and reduce the risk of overbuying for some buyers, including L&MICs, and increase equity.

### Transparency

**Fig. 2:**
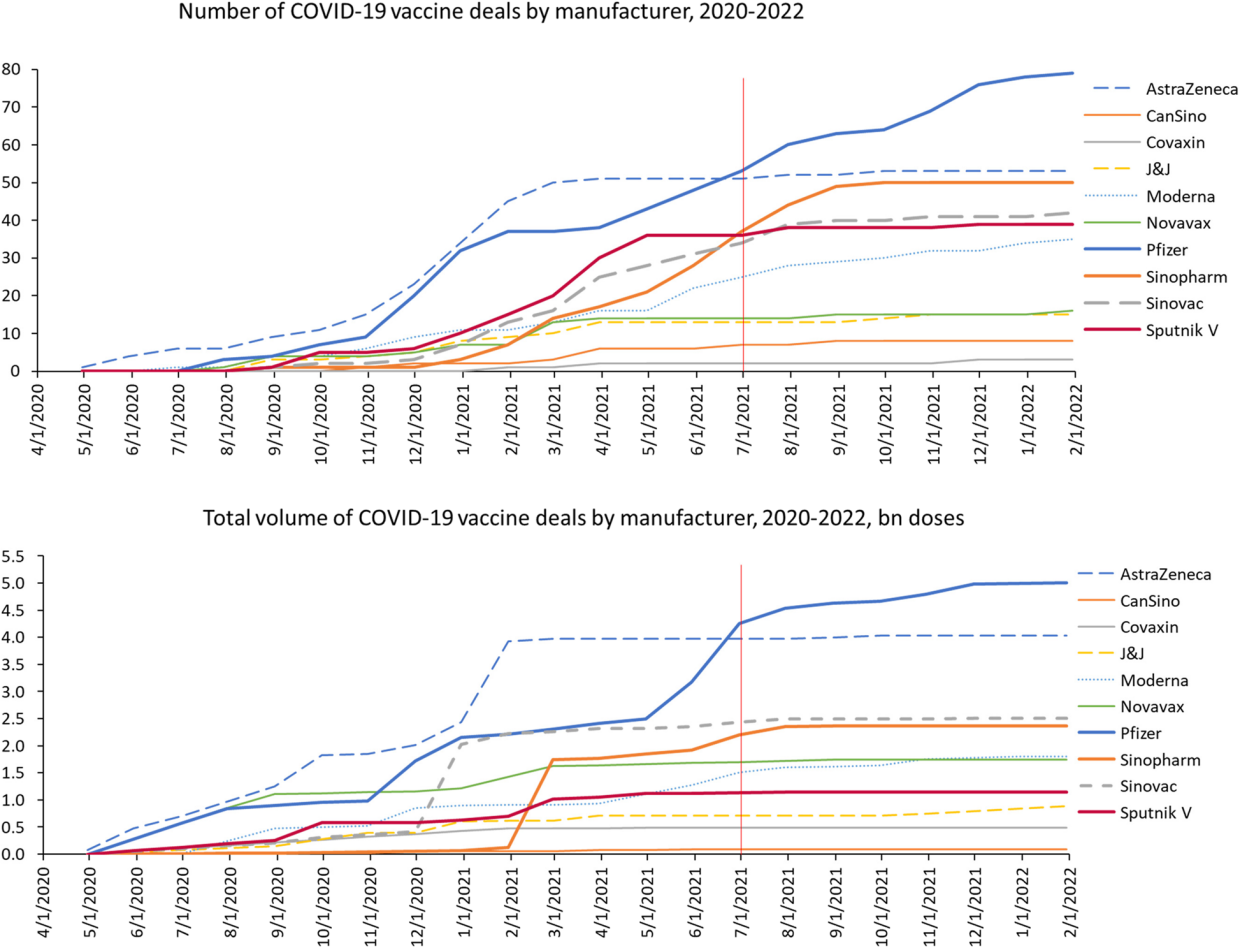
Ongoing dealmaking (number of deals, and doses) in COVID-19 Vaccines by manufacturer. The data on secured doses are from the UNICEF COVID-19 Vaccine Market Dashboard [50]

Greater market transparency might help buyers sign better deals and reduce the risk of aggregate overbuying. Market brokers could aggregate and publish available information on deals, which might help buyers to better assess the to what extent supply has been secured and likely timelines for their own desired products. Given the great uncertainties facing buyers in pandemics, we have to be realistic about what transparency can achieve, but timely, well-presented information can still act as a useful check, especially at later stages. In the case of COVID-19 vaccines, it was clear by June 2021 that the majority of R&D risk had passed [49] and that the 16 billion doses on order were already far above likely demand. Yet another 43 APAs for another 4 billion doses have been signed since then [50] (Fig. 2).

### Resale market

Resale markets could help distribute oversupply but are not guaranteed to improve equity or reduce overbuying. In a resale market, market forces would determine the prices for scarce versus abundant products, preferred versus non-preferred products, and short versus long shelf-life products. This could affect equity in either direction: HICs could buy up products in scarce supply, making them less accessible to L&MICs, or they could sell abundant products at deep discounts. Similarly, impacts on overbuying could go either way: essentially such a mechanism would allow buyers to choose their own balance of supply security/diversity versus cost. The resale market should have one advantage over donations, however: it should disincentivize the dumping of soon-to-expire or less desired products only when APA holders are absolutely sure they will not need them [51]. One potential challenge to this solution is the reluctance of some suppliers to allow secondary markets: it has been alleged that many COVID-19 vaccine suppliers prohibited resale of products within the terms of their APAs [52].

### Donation infrastructure

If donations are to remain the release valve for overbuying, future PPR efforts could build in infrastructure for this from the start. Donations of COVID-19 vaccines have been a cruder version of the resale solution proposed above. HICs that have overbought have donated bilaterally to L&MICs as well as through COVAX, the African Union, and/or UNICEF. As of early March 2022, twice as many of the doses that COVAX has delivered to L&MICs have come from donations (mostly from HICs) as have been purchased through APAs placed by COVAX [53, 54]. Donations were crucial when COVAX was struggling for supply in 2021 but have important drawbacks. Most importantly, the viability of donations as a way for L&MICs to obtain sufficient vaccines depends on HICs having excess supply —there is no guarantee that this will be the case in the next pandemic. Second, donations have allowed HICs to some extent to hide their overbuying (see Fig. 1), whereas COVAX and L&MICs more generally will end this year with millions of surplus doses at risk of wastage and no one to donate them to. Finally, the way that some vaccine donations have been valued in accounting for development aid—at a single price regardless of what the countries actually paid—risks allowing some donors to substitute artificially inflated donation credits for actual development expenditure [55]. Building mechanisms for donation into, or around, APAs could help accelerate and smooth the process [56], helping reduce inequity, though likely having limited impacts on overbuying.

### Transition away from APAs

Finally, buyers can begin to transition from APAs as pandemics and pandemic markets mature. This article argues that APAs, which insulate product developers from demand risk, make sense in the early stages of pandemic, when the course of the pandemic and thus product demand is highly unpredictable. But the logic of continuing to rely on this approach as a pandemic matures becomes less and less compelling, as R&D risk recedes, supply increases, and likely future demand is better understood. Buyers can reduce the risk of further overbuying by shifting demand risk back to suppliers, at least partially, and by introducing more competition into procurement. One way to do this within the context of bilateral deals with firms is to embody some of possible future demand in options, alongside some guaranteed volume—this practice is already being used by HIC buyers, COVAX and others. A second approach is to move away from bilateral APAs toward AMCs, which offer an assured market but not guaranteed sales to individual firms. Such AMCs can be designed to include competitive elements and pricing that reflects differential product performance [9, 57]. As the market matures further—for example, if a disease become endemic—and supply is no longer highly constrained, buyers can rely on competitive tendering – as is used for many other global health products.

These approaches could reduce overbuying and support equitable access. However, the uncertainty inherent in epidemics and the fact that others’ APAs tie up supply at the potential expense of yours can lead to a self-reinforcing APA cascade that can be difficult to escape.

These proposed mitigation measures/mechanisms vary in their likely utility for different types of buyers. Table 2 provides a subjective assessment of the seven measures/mechanisms described above by buyer type considering multiple factors (access, price, ability to use, necessary conditions for success).

**Table 2:** Assessment of mechanisms to mitigate the disadvantages of APAs

		**Mitigation Measure/Mechanism**						
		1.**Treaty**	2.**Pooling**	3.**Options fund/facility**	4.**Greater market transparency**	5.**Resale**	6.**Donation infrastructure**	7.**Transition away from APAs**
**How well does the measure/ mechanism work for each buyer type?**	**HIC state buyer**	**LOW** – most likely to ‘lose’ from an enforced treaty in terms of volume and speed of access	**HIGH** – HIC buyers can choose who they pool with and on what terms	**DEPENDS** on eligibility criteria. Not conceived as the primary channel of access for HICs	**MEDIUM**—HICs have greater capacity to use market information but probably already have greater access to this information	**HIGH** – have most buying power to use resale markets to secure if needed; and in face of surplus can recoup some expenditure	**MEDIUM** – can potentially claim ODA credits and otherwise gain soft power	**MEDIUM**—Can potentially reduce overbuying, but less to gain than other buyers less able to afford this risk
	**L&MIC state buyer**	**HIGH** – most likely to gain in terms of volume and speed of access, *if treaty provisions can be enforced*	**MEDIUM** – L&MIC buyers can choose who they pool with. But pooling cannot by itself compensate for shared lack of purchasing power	**MEDIUM** – L&MICs would still need up-front financing for the premium, and eventually the products. Most useful for MICs and for LICs if accompanied by donor subsidy	**LOW** – this is a weak lever and L&MICs have mixed capability to use the information to make better deals	**MEDIUM** – on average, these buyers tend to have lower buyer power for left-over doses, so only works well in situations of significant oversupply	**MEDIUM** – can help L&MICs plan and manage supply from different sources. But would remain dependent on HICs and benefit only if HICs have excess supply	**DEPENDS** on the context. Less use of APAs by others might allow L&MICs to secure supply, but also reduce donations from HICs
	**International organization buying on behalf of L&MICs**	**DEPENDS** on the content of the treaty and roles for international organizations in it, as well as on enforcement	N/A – international organizations are already pooling on behalf of L&MICs	**DEPENDS** on the interface between facility and existing international infrastructure. Call options could be used in COVAX’s ‘self-financing’ stream, the facility could use UNICEF to place deals, or it could be a completely sperate entity	**MEDIUM** –international procurement organizations are well placed to make good use of market information	**MEDIUM**/**HIGH** – with moderate buyer power and a diverse client base, should be able to use resale markets effectively	**DEPENDS** on whether donations flow through the international organization or outside it through bilateral arrangements with recipients	**HIGH** – can take advantage of flexibility to obtain better prices and reduce risk of overbuying in environment of adequate supply

## Conclusions

APAs are useful and will continue to be used in pandemics. Recent experience has demonstrated that APAs can be a powerful way to incentivize the development and manufacture of medical countermeasures, particularly in the context of disease outbreaks, when disease trajectory and epidemiology are highly unpredictable. From the perspective of HICs, APAs have been quite successful in recent pandemics, providing these countries with more assured access to H1N1 and COVID-19 vaccines. It is likely that these countries and other buyers with access to the necessary resources will continue to use these tools in future pandemics.

With no regrets purchasing, some *risk* of overbuying or overpaying is unavoidable – but that does not mean that overbuying/overpaying are forgone conclusions when APAs are deployed. APAs are useful because pandemics create an extraordinarily uncertain environment. This uncertainty means that buyers may have to buy product that they do not end up needing —if they, and suppliers, were sure that demand would materialize, an advance commitment to buy would not be necessary. Thus, the fact that a buyer is left with unneeded product does not necessarily mean that an APA was entered into in error, given what was known at the time. Ultimately, in the spirit of “no regrets”, in a pandemic too much supply is better than too little. While some risk of excess supply is unavoidable, it is of course possible to commit to buying too much or at too high a price. Crucially, however, the appropriate standard should be what was known *at the time* commitments were made, not what is known in hindsight. In the case of COVID-19 for example, no one could predict that such a high proportion of candidate vaccines would come to market.

The extensive use of APAs by HICs contributes to inequity in access by allowing these countries with greatest financial and technical capabilities to monopolize supply, at least in early stages. Although in the case of COVID-19 vaccines, HICs partially mitigated the impact of their deals with suppliers through donations, this occurred only after they had met their own domestic needs and did little to reduce inequity in the *timing* of access. If vaccine development had been less successful and fewer products had come to market, leaving supply persistently constrained, L&MICs might still be waiting for vaccines. While COVAX hoped to ensure supply for L&MICs through its own use of APAs, a variety of exogenous factors hampered its success (e.g., greater resources at the disposal of HICs combined with their ability to mobilize their funds and sign deals quickly; the use of trade restrictions by vaccine producing countries).

There are ways to reduce the equity impact of APAs while also mitigating some of the risks to buyers. Some of the measures/mechanisms outlined here, ranging from a pandemic treaty governing access to necessary medical countermeasures, to greater use of pooling and resale markets, to better planning for donations, could potentially help improve equity and reduce the risk of overbuying. At the same time, as a host of new PPR initiatives get off the ground in the wake of the COVID-19 pandemic, it will be important to be realistic about what these measures can achieve. Despite high aspirations, the APAs signed on behalf of L&MICs were not able to provide access to COVID-19 vaccines to many of these countries significantly earlier than donations from HICs or from India and China. Moreover, some of the proposed mitigation strategies outlined here were ultimately part of the COVID-19 response, yet the vaccine roll-out was nonetheless far less equitable than hoped. However, in many cases these measures were deployed late while others were discussed but not implemented, leaving grounds to hope that they could have a greater effect if incorporated in the response from the beginning. For the next global pandemic, the buying community focused on access for L&MICs should *accept as inevitable* the use of APAs by HICs and *focus on mitigating earlier and more systematically the resulting inequities*. Buyers should also consider in advance how and when to begin to shift demand risk back to suppliers, including through the greater use of options rather than purchase commitments and AMCs rather than bilateral APAs.

## Data Availability

The datasets analysed in Fig. 1 are available at https://www.imf.org/en/Topics/imf-and-covid19/IMF-WHO-COVID-19-Vaccine-Tracker and https://ourworldindata.org/covid-vaccinations. The datasets analysed in Fig. 2 are available in the UNICEF COVID-19 Vaccine Market Dashboard, https://www.unicef.org/supply/covid-19-vaccine-market-dashboard

## Abbreviations

AD: Auto Disable
ACT: Access to COVID-19 Tools
AMC: Advance Market Commitment
APA: Advance Purchase Agreement
APC: Advance Purchase Commitment
APPC: Advance Price or Purchase Commitment
COVID-19: Coronavirus Disease 2019
HICs: High Income Countries
L&MICs: Low- and Middle-Income Countries
PCV: Pneumococcal Conjugate Vaccine
PHEIC: Public Health Emergency of International Concern
POC: Point of Care
PPE: Personal Protective Equipment
PPR: Pandemic Preparedness & Response
RDT: Rapid Diagnostic Test
UMIC: Upper Middle-Income Country
UNICEF: United Nations Children's Fund
WHO: World Health Organization
ZIKV: Zika Virus

## References

[1] Jeremy Konyndyk, “It’s time for a ‘no regrets’ approach to coronavirus”. *The Washington Post*, 4th February 2020. Available at: https://www.washingtonpost.com/outlook/2020/02/04/its-time-no-regrets-approach-coronavirus/. Accessed on 14 Feb 2022.

[2] A Towse & H Kettler. (2005). “Advance price or purchase commitments to create markets for treatments for diseases of poverty: lessons from three policies.”. *Bulletin of the World Health Organization*, 83 (4), 301 - 307. World Health Organization. Accessed at: https://apps.who.int/iris/handle/10665/269378. Accessed 21 Feb 2022 PMC2626206 15868022

[3] Phelan AL, Eccleston-Turner M, Rourke M, Maleche A, Wang C (2020). Legal agreements: barriers and enablers to global equitable COVID-19 vaccine access. Lancet.

[4] Some actors distinguish between advance purchase ‘agreements’ and ‘contracts’ – with the latter considered to be legally binding. However, the term APA has been widely used in the context of pandemic response to refer to legally binding contractual instruments.

[5] S Jerving. ‘The fickle nature of COVID-19 vaccine agreements’. DEVEX. 06 May 2021. Available at: https://www.devex.com/news/the-fickle-nature-of-covid-19-vaccine-agreements-99826. Accessed on 8 March 2022.

[6] M. Kremer, A. Towse, & H. Williams. “Briefing Note on Advance Purchase Commitments”. DFID Health Systems Resource Centre. May 2005. Accessed at: https://www.who.int/intellectualproperty/submissions/MichealKremerKTW_CIPIH_submit_2.pdf?ua=1. Accessed on 15 Feb 2022.

[7] Agreements with this kind of conditionality on unpredictable external events can be thought of as “sleeping contracts”, as they may lie dormant for years.

[8] Susan Athey, Michael Kremer, Christopher Snyder and Alex Tabarrok. ‘In the Race for a Coronavirus Vaccine, We Must Go Big. Really, Really Big.’ New York Times. 04 May 2020. Available at: https://www.nytimes.com/2020/05/04/opinion/coronavirus-vaccine.html. Accessed on 07 March 2022.

[9] Towse A, Chalkidou K, Firth I, Kettler H, Silverman R (2021). How Should the World Pay for a Coronavirus Disease (COVID-19) Vaccine?. Value In Health.

[10] COVAX is global pandemic response collaboration, co-led by the Coalition for Epidemic Preparedness Innovations (CEPI), Gavi-The Vaccine Alline, and the World Health Organization (WHO), alongside key delivery partner UNICEF. Its aim is to accelerate the development and manufacture of COVID-19 vaccines, and to guarantee fair and equitable access for every country in the world.

[11] It is also possible that reducing demand risk for a particular product could accelerate the development of that product whilst in the pipeline. While firm-level decisions on R&D/production investment are complex and almost always confidential, having finance up front, as well as trust in a future market, may encourage businesses to prioritise a product within their portfolios for potential investment. If financing or prioritization were the rate limiting steps (rather than regulatory hurdles, say) then an APA could accelerate access.

[12] Contracting modalities of various 'firmnesses’ exist, such as Long-Term Agreements, Advance Market Commitments, and others, but if these are not always sufficiently robust or fit for purpose, an APA can legally ‘guarantee’ supply. To what extent APAs ‘guarantee’ supply can be debated. For example, many of the COVAX APAs guaranteed volumes of supply, but not hard delivery dates, which led to suspicions higher paying customers were being prioritized within available production capacity. On the other side Mark Turner (2016) ‘Vaccine procurement during an influenza pandemic and the role of Advance Purchase Agreements: Lessons from 2009-H1N1’, published in Global Public Health, 11:3,322–335, DOI: 10.1080/17441692.2015.1043743 show that customers who have overbought can often negotiate out of ‘legally binding’ APAs – something which must be considered in the risk-reward assumptions on both sides of the deal. 10.1080/17441692.2015.1043743 26209064

[13] By guaranteeing revenue and some market share, offering APAs to multiple firms can encourage more developers than might otherwise pursue a particular product to keep going. Given the high overall dropout rate in a product development process, this can also have the overall effect of accelerating development of a product in that category, even if it does not accelerate the R&D on individual products (see above).

[14] Influenza A virus subtype H5N1

[15] Turner M (2016). Vaccine procurement during an influenza pandemic and the role of Advance Purchase Agreements: Lessons from 2009–H1N1. Glob Public Health.

[16] Collin N, de Radiguès X (2009). Vaccine production capacity for seasonal and pandemic (H1N1) 2009 influenza. Vaccine.

[17] The ZIKV outbreak of 2015–2016 was initially concentrated in the Americas. In general, the region is characterized by robust surveillance systems including high coverage of fixed site confirmatory testing infrastructure. Accordingly, there was not a large need for POC RDTs across the region. However, the disease’s trajectory was uncertain, and no one was sure whether there would need to be a response to track ZIKV infections in countries with weak surveillance systems; for example, in parts of Africa and Asia where the *Aedes aegypti* mosquito responsible for spreading ZIKV was also present.

[18] UNICEF Evaluation Office. ‘Innovation Case studies: Zika Virus Diagnostics for Testing at Point-of-Care.’ November 2019. Accessed at: https://www.unicef.org/evaluation/media/986/file/Zika%20Virus%20Diagnostics%20for%20Testing%20at%20Point-of-Care.pdf

[19] Paul A. Wilson, May. Chu, Kristoffer. Gandrup-Marino, Nagwa. Hasanin, Philipp. Kalpaxis, Priya. Sharma, Lama. Suleiman, Jonathan. Weiss and Gian. Gandhi. ‘UNICEF’s Advance Purchase Commitment for Zika Virus diagnostic tests: design, results, and implications for other products’. *Submitted for publication*. Available on request.

[20] Richard G. Frank Leslie Dach & Nicole Lurie. ‘It Was The Government That Produced COVID-19 Vaccine Success.’ *Health Affairs*. 14 May 2021. Available at https://www.healthaffairs.org/do/10.1377/forefront.20210512.191448https://www.healthaffairs.org/do/10.1377/forefront.20210512.191448. Accessed on 16 Feb 2022.

[21] $5.0bn from Allied Market Research, https://www.alliedmarketresearch.com/influenza-vaccines-market Accessed 9 March 2022, $5.67bn from Verified Market Research https://www.verifiedmarketresearch.com/product/influenza-vaccine-market/ Accessed 9 March 2022. Accessed 9 March 2022

[22] Accelerating vaccine trials. (2021). Bulletin of the World Health Organization, 99(7), 482–483. 10.2471/BLT.21.020721 10.2471/BLT.21.020721 PMC8243025 34248219

[23] For example, the Bill and Melinda Gates Foundation has provided $150 m to Gavi, which in turn passed those on to the Serum Institute of India, providing it with “upfront capital” to help it increase manufacturing capacity for AstraZeneca and Novavax vaccines, even before regulatory approval and WHO prequalification had been obtained. https://www.gavi.org/news/media-room/new-collaboration-makes-further-100-million-doses-covid-19-vaccine-available-low

[24] Lythgoe MP, Middleton P. ‘Comparison of COVID-19 Vaccine Approvals at the US Food and Drug Administration, European Medicines Agency, and Health Canada.’ JAMA Netw Open. 2021;4(6):e2114531. 10.1001/jamanetworkopen.2021.14531. Accessed at: https://jamanetwork.com/journals/jamanetworkopen/fullarticle/2781352 10.1001/jamanetworkopen.2021.14531 PMC8233699 34170306

[25] Not all UNICEF purchasing of PPE was through APAs – these were used for about ~10% of the volumes purchased, to support capacity expansion (as listed in the main body of the paper) and to de-risk production for manufacturers who were new to dealing with UNICEF/UN procurement

[26] UNICEF Supply. ‘COVID-19 impact assessment and outlook on personal protective equipment’. 04 May 2020. Available at: https://www.unicef.org/supply/stories/covid-19-impact-assessment-and-outlook-personal-protective-equipment. Accessed on 16 Feb 2022.

[27] The RECOVERY Collaborative Group. ‘Dexamethasone in Hospitalized Patients with Covid-19.’ NEJM, Vol. 384(8), pp 693–704. 10.1056/NEJMoa2021436 10.1056/NEJMoa2021436 PMC7383595 32678530

[28] ACT-Accelerator moves to expand access to dexamethasone for low- and middle-income countries for COVID-19 treatment. 02 July 2020. Available at: https://unitaid.org/news-blog/act-accelerator-moves-to-expand-access-to-dexamethasone-for-low-and-middle-income-countries-for-covid-19-treatment/#en. Accessed on 17 Feb 2022.

[29] UNICEF Supply Division. “Report on the Procurement of health products in support of the ‘Access to COVID-19 Tools Accelerator -ACT-A)”. Available at: https://www.unicef.org/supply/media/8291/file/Dexamethasone-Inventory-August-2020-November-2021.pdf. Accessed on 02 March 2022.

[30] UNICEF internal management data, accurate as of 31 March 2022

[31] T Braithwaite. ‘We are drowning in vaccine.’ What will revive the market for Covid jabs?. Moderna’s chief says there is ‘massive oversupply’. Financial times, 22 April 2022. Available at: https://www.ft.com/content/dc35dfc0-94a2-440b-a880-91ba2e39c256. Accessed on 23 April 2022

[32] C Kay, M Kaur Makol, and J Paton. ‘World Moves From Shortages to Possible Glut of Covid-19 Vaccines’. Bloomberg. March 28, 2022, 5:00 PM EDT. Updated on March 29, 2022, 5:29 AM EDT. Available at: https://www.bloomberg.com/news/articles/2022-03-28/world-moves-from-shortages-to-possible-glut-of-covid-19-vaccines. Accessed on 30 March 2022.

[33] P Sanjai and Bloomberg. ‘The world’s biggest vaccine manufacturer has stopped making COVID jabs amid a 200 million dose glut’. FORTUNE. April 22, 2022 5:07 AM EDT. Available at: https://fortune.com/2022/04/22/serum-institute-world-biggest-vaccine-manufacturer-stopped-covid-jabs-200-million-dose-glut/. Accessed on 25 April 2022

[34] M Mishra and M Erman. ‘J&J pulls COVID vaccine sales forecast due to low demand, supply glut’. Reuters. April 19, 2022 5:31 PM EDT. Available at: https://www.reuters.com/business/johnson-johnson-suspends-sales-forecast-covid-19-vaccine-2022-04-19/. Accessed on 25 April 2022

[35] Cnops L, De Smet B, Mbala-Kingebeni P, van Griensven J, Ahuka-Mundeke S, Ariën KK (2019). Where are the Ebola diagnostics from last time?. Nature.

[36] Kameda K, Kelly AH, Lezaun J, Löwy I (2021). Imperfect diagnosis: The truncated legacies of Zika testing. Soc Stud Sci.

[37] SARS-CoV-2 infection in Africa: A systematic review and meta-analysis of standardised seroprevalence studies, from January 2020 to December 2021. HC Lewis, H Ware, M Whelan, L Subissi, Z Li, X Ma, A Nardone, M Valenciano, B Cheng, K Noel, C Cao, M Yanes-Lane, B Herring, A Talisuna, N Nsenga, T Balde, DA Clifton, M Van Kerkhove, DL Buckeridge, N Bobrovitz, J Okeibunor, RK Arora, I Bergeri, the UNITY Studies Collaborator Group medRxiv 2022.02.14.22270934; doi: 10.1101/2022.02.14.22270934 Accessed 7 March 2022 10.1136/bmjgh-2022-008793 PMC9402450 35998978

[38] Ewen. Callaway. ‘The unequal scramble for coronavirus vaccines — by the numbers’. *Nature*. NEWS EXPLAINER, 24 August 2020. Available at: https://www.nature.com/articles/d41586-020-02450-x. Accessed on 16 Feb 2022 10.1038/d41586-020-02450-x 32839593

[39] https://www.imf.org/en/Topics/imf-and-covid19/IMF-WHO-COVID-19-Vaccine-Tracker Accessed 28 March 2022

[40] https://ourworldindata.org/covid-vaccinations Accessed 28 March 2022

[41] European Council of the European Union. ‘An international treaty on pandemic prevention and preparedness’. Available at: https://www.consilium.europa.eu/en/policies/coronavirus/pandemic-treaty/. Accessed on 6 March 2022.

[42] World Health Organization (WHO). ‘World Health Assembly agrees to launch process to develop historic global accord on pandemic prevention, preparedness and response’. 1 December 2021. Available at: https://www.who.int/news/item/01-12-2021-world-health-assembly-agrees-to-launch-process-to-develop-historic-global-accord-on-pandemic-prevention-preparedness-and-response. Accessed on 6 March 2022.

[43] I. Kickbusch & A. Holzscheiter. ‘Can geopolitics derail the pandemic treaty?’. BMJ 2021; 375 doi: 10.1136/bmj-2021-069129 10.1136/bmj-2021-069129 PMC8621752 34836885

[44] D.P. Fidler. ‘The Case Against A Pandemic Treaty’. Think Global Health. 26 November 2021. Available at: https://www.thinkglobalhealth.org/article/case-against-pandemic-treaty. Accessed on 6 March 2022.

[45] Bollyky TJ, Gostin LO, Hamburg MA (2020). The Equitable Distribution of COVID-19 Therapeutics and Vaccines. JAMA.

[46] Stein F (2021). Risky business: COVAX and the financialization of global vaccine equity. Global Health.

[47] https://ec.europa.eu/info/live-work-travel-eu/coronavirus-response/public-health/eu-vaccines-strategy_en Accessed 23 March 2022

[48] See https://www.tcxfund.com/about-the-fund/ for example

[49] At this point, Pfizer-BioNTech, Moderna, AZ, Johnson & Johnson/Janssen, Sinovac and Sinopharm had all been approved by the WHO or a Stringent Regulatory Authority.

[50] UNICEF Vaccine Market Dashboard, https://www.unicef.org/supply/covid-19-vaccine-market-dashboard

[51] de Bengy Puyvallée, A., Storeng, K.T. COVAX, vaccine donations and the politics of global vaccine inequity. *Global Health* 18, 26 (2022). 10.1186/s12992-022-00801-z 10.1186/s12992-022-00801-z PMC8897760 35248116

[52] M. Apuzzo and& S. Gebrekidan. ‘Governments Sign Secret Vaccine Deals. Here’s What They Hide.’ New York Times. Published Jan. 28, 2021 and Updated Feb. 24, 2021. Available at: https://www.nytimes.com/2021/01/28/world/europe/vaccine-secret-contracts-prices.html. Accessed on 6 March 2022.

[53] UNICEF Market Dashboard, 9 March 2022. AMC92 countries. Donated and facilitated deliveries ~734m, APAs at ~347m, and cost shared doses at ~113m.

[54] A Puyvallée & K Storeng. ‘COVAX, vaccine donations and the politics of global vaccine inequity’. Globalization and Health. Vol 18(26). 2022. 10.1186/s12992-022-00801-z 10.1186/s12992-022-00801-z PMC8897760 35248116

[55] https://www.cgdev.org/blog/vaccine-mark-counting-more-oda-we-paid-vaccines-illogical-immoral-and-unpopular Accessed 23 March 2022

[56] This could include demand forecasting from potential recipients, the donor-recipient matching facility, country readiness support for those donations, ex ante discussion of ODA treatment, etc.

[57] Kremer, Michael and Levin, Jonathan D. and Snyder, Christopher M., Designing Advance Market Commitments for New Vaccines (December 2020). NBER Working Paper No. w28168, Available at SSRN: https://ssrn.com/abstract=3743901 Accessed 28 March 2022

